# Music Training, and the Ability of Musicians to Harmonize, Are Associated With Enhanced Planning and Problem-Solving

**DOI:** 10.3389/fpsyg.2021.805186

**Published:** 2022-01-27

**Authors:** Jenna L. Winston, Barbara M. Jazwinski, David M. Corey, Paul J. Colombo

**Affiliations:** ^1^Department of Psychology, Tulane University, New Orleans, LA, United States; ^2^Department of Music, Tulane University, New Orleans, LA, United States; ^3^Brain Institute, Tulane University, New Orleans, LA, United States

**Keywords:** music training, executive function, musicians, planning, harmony, cognition, problem solving

## Abstract

Music training is associated with enhanced executive function but little is known about the extent to which harmonic aspects of musical training are associated with components of executive function. In the current study, an array of cognitive tests associated with one or more components of executive function, was administered to young adult musicians and non-musicians. To investigate how harmonic aspects of musical training relate to executive function, a test of the ability to compose a four-part harmony was developed and administered to musicians. We tested the working hypothesis that musicians would outperform non-musicians on measures of executive function, and that among musicians, the ability to harmonize would correlate positively with measures of executive function. Results indicate that musicians outperformed non-musicians on the Tower of London task, a measure of planning and problem-solving. Group differences were not detected on tasks more selective for inhibitory control, conflict resolution, or working memory. Among musicians, scores on the harmony assessment were positively correlated with performance of the Tower of London task. Taken together, the current results support a strong relationship between musicianship and planning and problem solving abilities, and indicate that the ability to harmonize is associated with components of executive function contributing to planning and problem solving.

## Introduction

Practicing, learning, or performing music engages a wide range of higher-order cognitive processes (Zatorre and McGill, 2005), induces neural plasticity (Kraus et al., 2009; Zendel and Alain, 2013; Schroeder et al., 2016; Sachs et al., 2017; Hennessy et al., 2019) and improves performance on a wide array of cognitive tasks used to measure executive function (Moreno et al., 2009, 2011; Zuk et al., 2014; Okada and Slevc, 2018). While there is strong evidence for a positive relationship between music and cognition, the causality of these effects, genetic predispositions, and motivation to pursue music remain controversial (see Sala and Gobet, 2020; Bigand and Tillmann, 2021 for reviews). Nevertheless, support remains for the use of music training to investigate experience-dependent changes in human cognition.

Executive function is a set of higher order mental processes that allow for self-regulation and goal-directed behavior (Miyake et al., 2000). Executive function is reportedly comprised of at least three core components: inhibitory control, working memory, and cognitive flexibility (Miyake et al., 2000; Lehto et al., 2003; Diamond, 2013; Zelazo, 2015). Inhibitory control is the ability to control thoughts, emotions, and behaviors by suppressing a habitual or automatic response. Working memory refers to time- and capacity-limited stores of task-specific information. Cognitive flexibility is the ability to successfully shift between task demands or mental sets. Higher-order cognitive functions built upon this core include selective attention, planning, and problem-solving (Collins and Koechlin, 2012; Lunt et al., 2012). The use of executive function is effortful, requires attentional resources and cognitive control, and is necessary for a wide range of daily activities that require regulation of thoughts and behavior (Miyake and Friedman, 2012). The importance of executive function in our lives is perhaps best illustrated by how well they predict long-term achievement. For example, executive function is reportedly more predictive of academic readiness than intelligence (Blair and Razza, 2007) and can predict reading and math abilities (Gathercole et al., 2004) and academic achievement (Best et al., 2011) at all grade levels. In adults, measures of executive function are positively correlated with job success (Bailey, 2007), marital satisfaction (Eakin et al., 2004), and overall quality of life (Brown and Landgraf, 2010; Davis et al., 2010).

Efforts to understand relationships between music training, which may include formal music lessons or long-term commitment to practicing and improving at least one musical instrument, and executive function have increased in recent years. Reports indicate that both child and adult musicians outperform non-musicians on tasks related to each component of executive function, including: (1) Working memory- measured by the backwards digit span (Zuk et al., 2014; Clayton et al., 2016) and n-back (Hou et al., 2014; Slevc et al., 2016) tasks. (2) Cognitive flexibility—measured by word fluency (Zuk et al., 2014; Janus et al., 2016), design fluency (Zuk et al., 2014), trail-making (Thaut et al., 2009; Zuk et al., 2014; Saarikivi et al., 2019), and task switching (Moradzadeh et al., 2014). (3) Inhibitory control—measured by the Simon (Bialystok and Depape, 2009; Amer et al., 2013; Joret et al., 2017), Stroop (Bialystok and Depape, 2009; Travis et al., 2011) and Go/No-Go tasks (Moreno et al., 2011, 2014). Of importance, studies that include random assignment to music training and active control groups suggest the musician benefit to executive function may result from training. For example, Moreno et al. (2011) compared inhibitory control and event-related potentials (ERPs) in children before and after 20 days of either music instruction or commensurate visual arts instruction. Children were matched for age, IQ, and parental education, and randomly assigned to either group. The researchers found that only the music group demonstrated significant improvements in behavioral performance on the go/no-go task, accompanied by increased amplitude of the P2 ERP, a neural correlate of task performance. In addition, working memory was significantly improved in musically naïve older adults after sixteen weeks of piano training or percussion training, but not after sixteen weeks of a music listening intervention (Bugos, 2019). This finding is convergent with earlier reports of improved executive function in older adults after 6 months of piano training (Bugos et al., 2007).

The examples cited above provide strong evidence that music training enhances executive function, but there are also reports that musicians do not always outperform non-musicians on these tasks. For example, Slevc et al. (2016) reported that musicians and non-musicians performed equivalently at task-switching, and musicians were worse than non-musicians on a color-word Stroop task. In addition, musicians and non-musicians did not differ in behavioral performance on a Stroop task, despite group differences for functional brain networks involved (Sachs et al., 2017; Hennessy et al., 2019). Recent meta-analyses on cognitive training programs have even suggested that human cognition may not be susceptible to far transfer effects, which are improvements in skills unrelated to training (see Sala et al., 2019 for review). Accordingly, a recent meta-analysis of studies that have shown music training effects in children concluded that the overall effect of music training is null, and that emphasis on the advantages of musical training may be misinterpretations (Sala and Gobet, 2020). This statement was based on two main conclusions: (1) when only randomized studies were considered, such that confounding variables could not influence the outcome, the effect of music training on cognition and academic achievement was null, and (2) the effect of music training was also null for studies using an active control group. Indeed, an alternative explanation for observed far transfer effects may be that more cognitively advanced people are predisposed to select and succeed at music (Schellenberg, 2020). However, other authors have criticized the methods used in Sala and Gobet (2020) meta-analysis. Bigand and Tillmann (2021) re-analyzed the same dataset, and concluded that far transfer effects of music training may have been underestimated by Sala and Gobet (2020). When randomization was not treated as a moderator and near transfer effects of active controls were neutralized, the same dataset provided evidence for a positive effect of music training on general cognition. These conclusions are convergent with other recent meta-analyses that show a significant effect of music training on cognition (Talamini et al., 2017; Cooper, 2020; Román-Caballero et al., 2021). Despite the ongoing debate of the relationship between music training and cognition, all authors agree that the question of causality remains, and cannot be definitively answered without new studies that rigorously control for randomization, fairly matched control groups, and the inclusion of pre and post-test measurements.

Further divergence in effects of music training may be attributable to differences in the criteria used to define “musicians,” and the type and extent of music training received by participants. For example, Slevc et al. (2016) did not find an effect of musicianship on inhibitory control in young adults, whereas previous reports have (Bialystok and Depape, 2009; Travis et al., 2011; Moreno et al., 2014). Slevc et al. (2016) defined musicianship by a combination of musical ability, measured by scores on the Musical Ear Test (MET), and musical experience, measured by the Ollen Musical Sophistication Index (OMSI). Others who report a positive association between musicianship and inhibitory control in young adults have defined musicianship as the number of hours of daily practice and context of music instruction (Bialystok and Depape, 2009), whether one is a professional or amateur musician (Travis et al., 2011), and six or more years of continuous Western classical training on a primary instrument (Moreno et al., 2014). A better understanding is likely to result from testing how components of music training, such as rhythm and harmony, are related to executive function. For example, percussionists show advantages in both auditory processing of rhythm, and inhibitory control, in comparisons with other types of musicians and non-musicians (Slater and Kraus, 2016; Slater et al., 2018). Another group tested whether pitch-based or rhythm-based music training would lead to differential outcomes in pre-school children. In line with previous findings, the authors reported that those in the rhythm group outperformed the pitch group and non-musical controls on measures of inhibitory control (Frischen et al., 2019). These reports suggest that rhythmic ability is more selectively related to inhibitory control than to working memory or cognitive flexibility. Further, these findings provide evidence that certain types of musical training may influence particular components of executive function and not others. While this approach has furthered our understanding of rhythm and executive function, another musical practice that may selectively relate to executive functions is harmonization.

Harmonization, in this context, refers to the ability to add complimentary notes to an existing melody in order to produce harmony. Completing a harmonization requires complex regulation of one’s thoughts and actions within a musical context. One must consider how any given note will influence the design of the piece, including the tension and resolution occurring as the piece moves through time. The most obvious answer will rarely be the most creative or satisfactory for the piece as a whole. Therefore, choosing notes best suited to the piece requires inhibitory control. Furthermore, because functional roles of musical notes are transformed by their harmonic and melodic context, one must frequently shift attention both horizontally (as the melody unfolds over time) and vertically (in harmony with other voices at a single point in time). The ability to view the piece from these varying perspectives, and to switch between them, requires cognitive flexibility. Harmonization is also built on learned rules and conventions, similar to grammar in a spoken language, which the composer must rapidly recall and apply to the current context. These processes challenge the capacity and duration of working memory. Thus the task demands of harmonization likely require inhibitory control, cognitive flexibility, and working memory, which together comprise the core of executive function. Moreover, harmonization may require coordination of these functions for selective attention, planning, and problem-solving, since all three core components are required in parallel. The practice of harmonization, therefore, may serve as a method for strengthening executive function. To our knowledge, little is known regarding the relationship between the ability to harmonize and executive functions.

The aims of the current study are to (1) test for an association between formal music training and performance on tests of executive function, and (2) examine, within musicians, whether there is an association between performance on tests of executive function and the ability to create a four-part harmonization. We hypothesize that (1) musical training is positively associated with measures of working memory, inhibitory control, and cognitive flexibility, and (2) among musicians, the ability to harmonize is positively associated with these measures as well.

## Materials and Methods

### Participants

Eighty-eight English-speaking undergraduate students (49 females, 39 males, mean age = 19.31) at Tulane University participated in the study. Musically experienced participants were recruited from undergraduate harmony courses, and musically inexperienced participants were recruited through Tulane University’s experiment management system.

### Musical Scale Variable

To measure musical experience, a musicianship scale continuous variable was constructed using principal component analysis. Scale construction was based on responses to eight questions (Golob et al., 2017) regarding musical experience. The variables included were: importance of music in the participant’s daily life (self-rated 1–10), number of first degree relatives who are professional musicians (Nrelatives), number of instruments played (Ninstruments), total years of musical experience, years of musical experience in which the participant played consistently for at least 3 h per week, average hours of playing per week, years of formal training, and ability to sight-read music (self-rated 1–10). These items were included in the scale because for each item, there was a numeric value for every participant, even if they had no musical experience.

**FIGURE 1:**
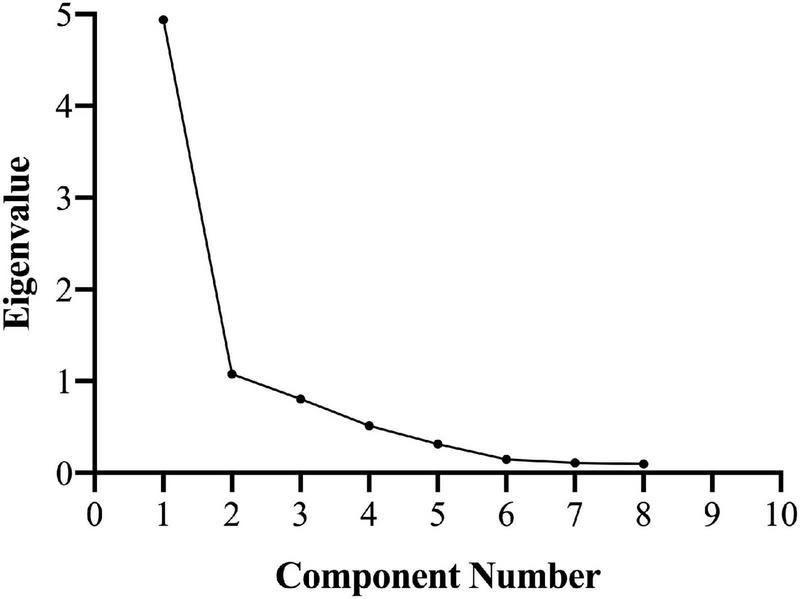
Scree plot resulting from 8 musicianship variables.

Each item was correlated with at least one other at a value greater than 0.3 [see Table 1; KMO = 0.838, χ_(28)^2^ = 208.08, *p* < 0.001] suggesting reasonable factorability. Inspection of the scree plot indicated a single factor (see Figure 1), onto which all variables loaded high (Importance = 0.539, Nrelatives = 0.584, Ninstruments = 0.826, Years Experience = 0.914, Years Instrument 1 = 0.826, Hours Per Week = 0.654, Years Formal Training = 0.901, Reading Ability = 0.929). This component explained 61.72% of the total variance in scores. The factor loading matrix for the single component is presented in Table 2. Internal consistency for the 8-item scale was strong (α = 0.902).

**TABLE 1:** Correlations among musicianship variables.

		Importance of music	# Pro. musician relatives	# Instruments played	Years of musical experience	Years on primary instrument	Average hours of practice per week	Years of formal training	Sight-reading ability
Importance of music	*r*	1	0.381	0.376	0.470	0.461	0.269	0.427	0.443
	*p*		0.015	0.017	0.002	0.004	0.093	0.007	0.005

# Professional musician relatives	*r*	0.381	1	0.446	0.462	0.214	0.431	0.544	0.377
	*p*	0.015		0.004	0.003	0.204	0.006	0.000	0.018

# Instruments played	*r*	0.376	0.446	1	0.619	0.597	0.631	0.618	0.684
	*p*	0.017	0.004		0.000	0.000	0.000	0.000	0.000

Years of musical experience	*r*	0.470	0.462	0.619	1	0.860	0.516	0.803	0.758
	*p*	0.002	0.003	0.000		0.000	0.000	0.000	0.000

Years on primary instrument	*r*	0.461	0.214	0.597	0.860	1	0.464	0.719	0.774
	*p*	0.004	0.204	0.000	0.000		0.000	0.000	0.000

Average hours of practice per week	*r*	0.269	0.431	0.631	0.516	0.464	1	0.481	0.553
	*p*	0.093	0.006	0.000	0.000	0.000		0.000	0.000

Years of formal training	*r*	0.427	0.544	0.618	0.803	0.719	0.481	1	0.702
	*p*	0.007	0.000	0.000	0.000	0.000	0.000		0.000

Sight-reading ability	*r*	0.443	0.377	0.684	0.758	0.774	0.553	0.702	1
	*p*	0.005	0.018	0.000	0.000	0.000	0.000	0.000	

**TABLE 2:** Factor loading matrix for the single component derived from the 8-item scale.

	Component 1
Importance of music	0.539
# Professional musician relatives	0.584
# Instruments played	0.826
Years of musical experience	0.914
Years on primary instrument	0.826
Average hours of practice per week	0.654
Years of formal training	0.901
Sight-reading ability	0.929

### Assessment of Executive Function

All participants completed a computerized battery of cognitive tests commonly used to measure EF including the Go/No-Go, Stroop, Simon task, Tower of London, and the Backwards Digit Span. All tasks were accessed through the Psychology Experiment Building Language (PEBL) software and administered without modification. Psychometric properties of each test were assessed in a normative sample in the same age range as the current sample (18–22 years), and validity and reliability were in a suitable range for basic research (Piper et al., 2015). A description of each task and the component(s) of executive function measured follows:

(a) Go/No-Go

The go/no-go task measures inhibitory control in a stop/go paradigm (Mueller and Piper, 2014). Participants were presented with either a P or an R, and instructed to press a computer key only when presented with a P. P’s were presented for 80% of trials, in order to establish an expected key press response to a visual stimulus. The number of key presses to R (inhibition failures) was measured.

(b) Stroop

The Stroop task measures inhibitory control of attention by creating interference between a stimulus and its required response (Mueller and Piper, 2014). A numerical Stroop task was used, in which participants were presented with the numbers 1, 2, or 3 and instructed to press the number on the keyboard corresponding to the number of characters in each trial. For example, for “111,” the correct response would be 3, and for “3” the correct response would be “1.” Participants were presented with congruent and incongruent trials (“333” being congruent, “111” being incongruent), and the differences in accuracy and reaction time between the two conditions were used as dependent measures.

(c) Simon

The Simon task manipulates stimulus-response compatibility, requiring the participant to engage cognitive control and suppress impulsive responses in order to respond correctly (Mueller and Piper, 2014). Participants were presented with a blue or red circle, either of which could appear at various positions on an invisible number line across the screen (from far right to far left). Participants were instructed to press the left shift key for a blue circle, and the right shift key for a red circle. The difference in accuracy and reaction time between congruent (blue—left, red—right) and incongruent (blue—right, red—left) trials were the dependent measures.

(d) Tower of London

The Tower of London task assesses problem-solving and planning abilities. Participants were presented with two sets of three rings, one set was the “goal state,” and one set was the workspace (Mueller and Piper, 2014). Participants were instructed to move the rings in the workspace into the configuration shown in the goal state. Participants were allowed 3–4 moves per trial, depending on trial complexity, to get the rings into the goal state. The limit on moves was chosen to necessitate deliberate planning and problem-solving. The total number of problems solved was the dependent measure.

(e) Backwards Digit Span

The backwards digit span task measures working memory (Mueller and Piper, 2014). Participants were presented with a list of three numbers, asked to memorize the list, and then to type the list on the computer from memory in the opposite order in which it was presented. If the participant responded correctly, then the list length increased by one digit for each correct response. The maximum number of digits that the participant entered correctly served as the dependent measure.

### Assessment of Harmonic Ability

To assess harmonic ability, an eight measure melody was developed by one of the authors (B.J.), a prolific composer and professor of Composition at Tulane University. Intra- and inter-rater reliability were assessed by re-scoring 33 harmonizations by the original rater, and by a Composition professor unaffiliated with the study, respectively. Harmonizations were de-identified using participant ID codes, and were re-scored without access to the original scoring sheet. Participants were asked to attempt the harmonization exercise if they had 10 or more years of continuous musical experience. We chose this criterion because it would allow us to draw meaningful conclusions about effects seen within musicians, and because completing this harmonization assessment would require extensive knowledge of classical Western European harmonic practices that could only be attained through high-level training and experience. Musically experienced participants were given eight measures of a four-part staff (SATB), with the provided melody written into the soprano line, and the alto, tenor and bass lines left blank. They were instructed to harmonize the soprano line in four voices in accordance with the rules and conventions of Western European harmonic practice. Harmonizations were scored on the basis of six criteria: (1) creativity/imagination, (2) sophisticated solutions with regard to selections of specific harmonic structures that result in desired harmonic motion, (3) ability to construct balanced phrases characterized by a sense of motion and direction, (4) ability to shape interesting horizontal lines, (5) ability to interpret and to simultaneously handle multiple rules which, at times, may require exceptions or alternative solutions, (6) ability to recall multiple rules with regard to horizontal voice-leading, doubling spacing, and range.

**FIGURE 2:**
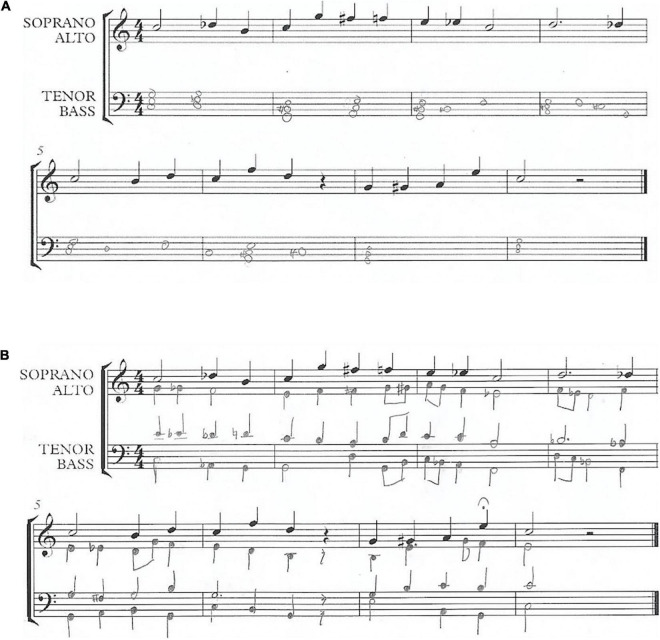
Typical low **(A)** and high **(B)** scoring examples of the harmonization task.

Responses were scored 0–4 for each of these six criteria as follows: Participants were scored a 0 = no effort and understanding of the concept; 1 = minimal effort was present but the concept was severely misunderstood or unrealized in the harmonization; 2 = some effort and understanding were present but parts were executed poorly or incorrectly; 3 = effort and understanding were present and the result was technically correct; 4 = effort and understanding were both maximally present and the result was both technically correct and musically exceptional. Accordingly, the highest score possible was 24, resulting from scoring a 4 in each area, and the lowest possible score was 0. Figure 2A shows an example of a low scoring harmonization, and Figure 2B shows an example of a high scoring harmonization.

The unique issue of operationalizing harmonic skill is that if every composer precisely followed all Western rules and conventions, developed in most cases for practical reasons, then all of their music would sound the same. For instance, one is not likely to find parallel fifths in a Bach composition, and the composer uses few augmented or diminished intervals. However, Debussy utilized them frequently to cultivate the style that made him an exceptional composer. Thus, simply knowing the rules and how to follow them is not all that is involved with harmonic skill. As the rules that govern harmonic practices continue to change throughout historical periods reflecting different cultures of thinking and setting different conventions as composers adapt the rules to suit their expressive needs. The criteria presented here were selected to not only highlight technical prowess, but also creativity and the capacity for innovation, which are essential for mastering harmonic composition.

### Procedure

After obtaining informed consent, participants completed the musical experience survey (Golob et al., 2017), then completed the cognitive task battery in a pseudo-random order. Tasks were completed individually, and the cognitive assessment lasted for 45 min. Participants who met the criteria described above for a highly experienced musician were asked to complete the harmonization assessment. Participants were given as long as they needed to complete the harmonization assessment, and completion times ranged from 5 to 75 min.

### Statistical Analysis

Pearson correlations were used to test associations between music scale score and scores on each cognitive measure. Pearson correlations were then used to test the associations between harmonization score and each cognitive measure that was significantly related to music scale score.

## Results

### Cognitive Evaluation

**FIGURE 3:**
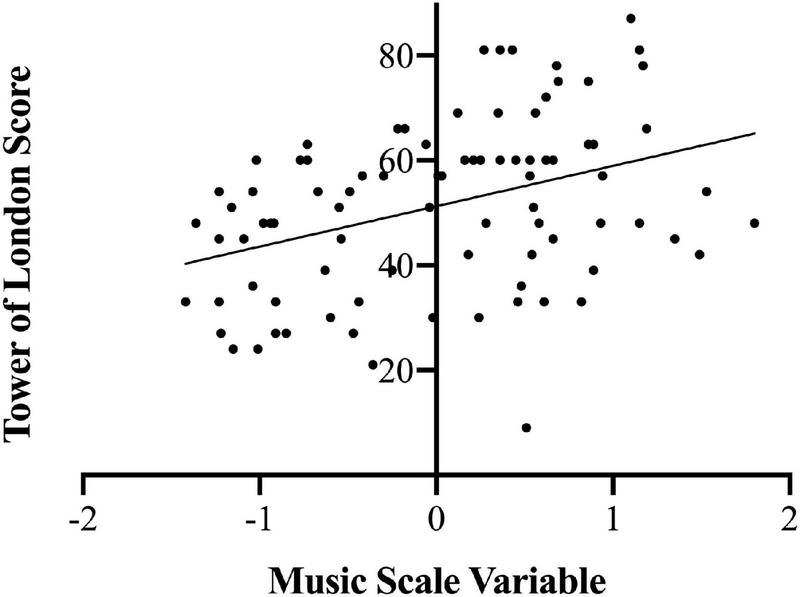
Correlation between scores on the Tower of London and music scale variable.

Table 3 shows descriptive statistics for all cognitive variables and the music scale variable. In order to test for an association between music training and executive function, Pearson correlations were run between the *Musical Scale Variable* (*MSV*) and scores on each cognitive measure. Significance levels were adjusted for multiple comparisons using Holm’s sequential Bonferroni adjustment (Holm, 1979). There was a significant positive correlation between *Tower of London Score* and *MSV* (*r* = 0.38, *p* = 0.002). This effect is illustrated in Figure 3. No other significant correlations were detected between *MSV* and other measures of executive function, including *Backward Digit Span* (*r* = 0.234, *p* = 0.182), *Go-No/Go* (*r* = 0.078, *p* = 0.476), *Stroop Task Accuracy* (*r* = −0.102, *p* = 0.351), *Stroop Task Reaction Time* (*r* = 0.016, *p* = 0.886), *Simon Task Accuracy* (*r* = 0.141, *p* = 0.191), and *Simon Task Reaction Time* (*r* = 0.152, *p* = 0.158).

**TABLE 3:** Descriptive statistics of cognitive variables, music scale variable, and harmony scores.

	N	Minimum	Maximum	Mean	Standard deviation
*Music scale variable*	88	–1.42	1.80	0.0291	0.81
*Harmony score*	52	0.00	24.00	11.52	6.02
*Backwards digit span*	86	3.00	10.00	7.15	1.80
*Go/No-Go inhibition failures*	85	1.00	27.00	10.20	5.53
Stroop effect -accuracy*	86	–0.06	0.39	0.10	0.08
Stroop effect—Median RT*	86	–227.50	–8.50	–72.61	38.56
Simon effect—Accuracy*	88	–0.05	0.33	0.04	0.05
Simon effect—Median RT*	88	–208.50	14.50	–37.66	31.56
*Tower of London score*	88	9.00	87.00	51.48	16.32

*Stroop and Simon Effects on accuracy and reaction time were calculated as Congruent—Incongruent.

**FIGURE 4:**
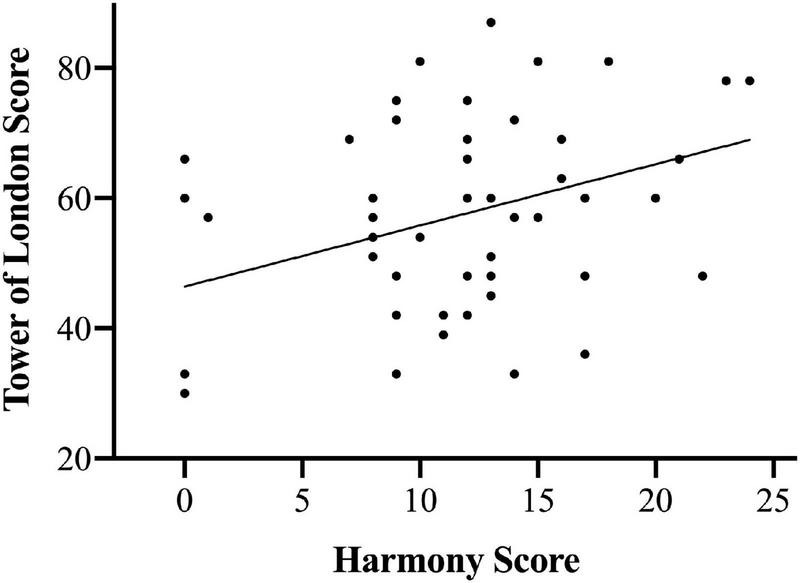
Correlation between scores on the Tower of London task and harmonization assessment.

In order to test for an association between the ability to harmonize and executive function, a Pearson correlation analysis was used to assess the relationship between *Harmony Score* and *Tower of London Score*. Results indicated a significant relationship between *Harmony Score* and *Tower of London Score* (*r* = 0.355, *p* = 0.010). This effect is illustrated in Figure 4. Correlations conducted between the *MSV* and demographic variables did not indicate relationships between *MSV* scores and *Sex* (*r* = 0.077, *p* = 0.477) or between *MSV* and *Age* (*r* = −0.003, *p* = 0.981). *Harmony Score* was also unrelated to *Sex* (*r* = −0.108, *p* = 0.445) and *Age* (*r* = −0.122, *p* = 0.390).

### Moderation Evaluation

In order to test whether *Harmony Score* moderated the relationship between *MSV* and *Tower of London Score*, ordinary least squares (OLS) moderated multiple linear regression was conducted. The regression analysis indicated that *Harmony Score* did not moderate the relationship between *Tower of London Score* and *MSV*, as the predictor of the interaction term *MSV × Harmony Score* was not significant (B 0.070, *p* = 0.748).

### Reliability Evaluation

**FIGURE 5:**
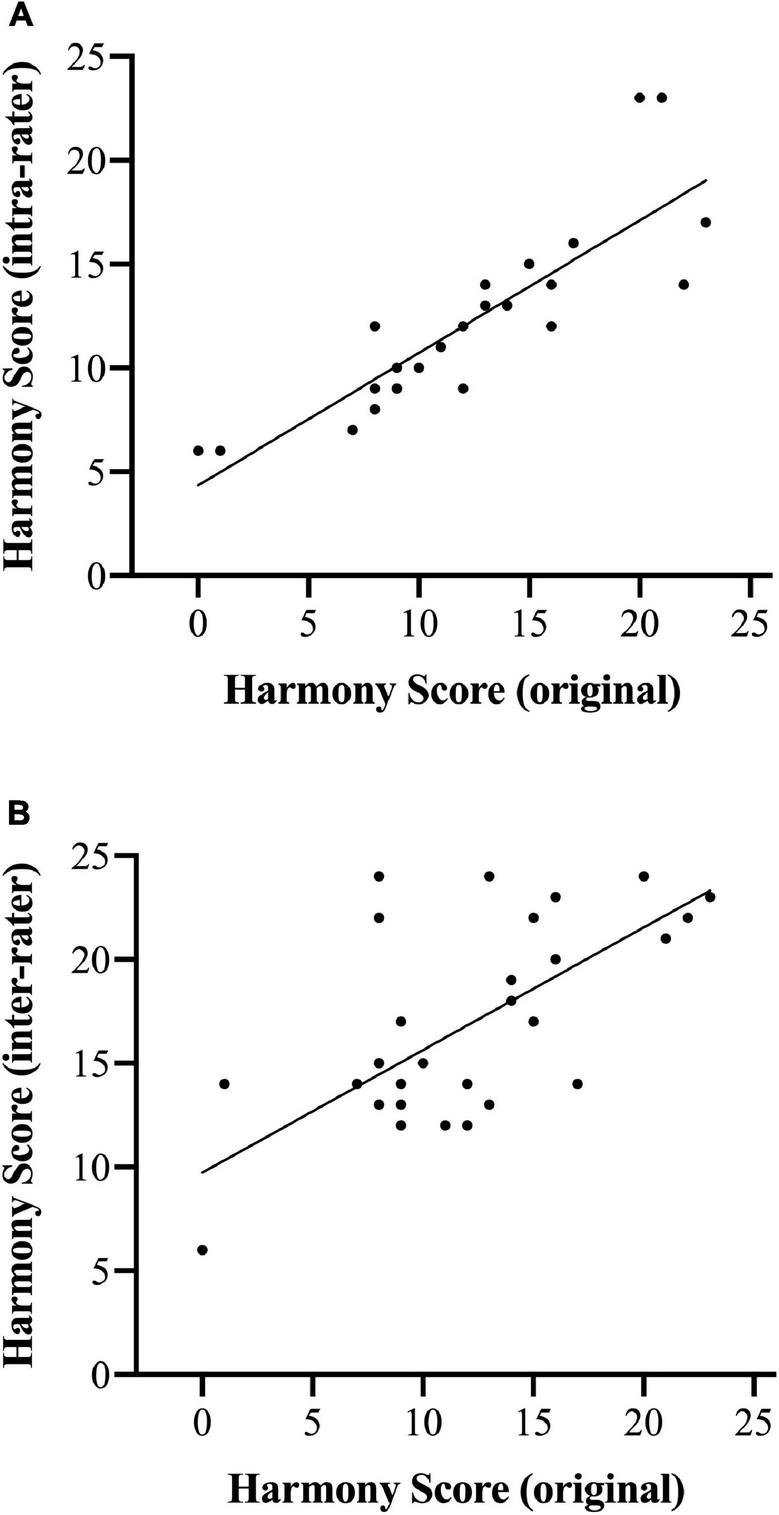
Correlations between original harmony scores and re-scores by the same rater **(A)** and a different rater **(B)**.

Participants who received a score of 0 for leaving their harmonization blank were omitted from the reliability analyses. There was strong intra-rater reliability indicated by the high correlation between the original and rescored values (*r* = 0.856, *p* < 0.001). This relationship is shown in Figure 5A. Harmonizations were also rescored by a composition professor who was unaffiliated with the study. Original harmony scores were positively correlated with re-scored values from the additional rater (*r* = 0.538, *p* = 0.004), suggesting high inter-rater reliability. This relationship is shown in Figure 5B.

## Discussion

The current study tested whether (1) music training is related to some or all components of executive function, and (2) among musicians, the ability to harmonize is associated with some or all executive functions. Results indicated that music training is positively associated with performance on the Tower of London task, a measure of planning and problem solving. Music training was not related to performance of the Backward Digit Span, Stroop, Simon, or Go/No-Go tasks. Among participants with high levels of musical experience, there is a positive association between harmony scores and scores on the Tower of London task. The current study also includes initial measures of reliability for a novel test used to quantify the ability to harmonize. While further validity and reliability is needed for the harmonization task used, these findings provide the first evidence relating the ability to harmonize to executive function.

Among musically experienced participants, the current results indicate that scores on the Tower of London, but not the other measures of executive function, were related to scores on the harmony assessment. Planning and problem-solving, which are central to both the Tower of London task and harmonization, are considered higher-order processes that emerge hierarchically from coordination of the core components of executive function (Collins and Koechlin, 2012; Lunt et al., 2012; Diamond, 2013). Indeed, successful completion of the Tower of London task requires coordination of inhibitory control to prevent an impulsive response before the correct path is planned, cognitive flexibility to imagine and select among alternative outcomes, and working memory to mentally retrace the sequence of moves. Successful harmonization also requires coordination of inhibitory control, cognitive flexibility, and working memory. Taken together, perhaps the process of coordinating the components of executive function may be a higher order property itself, dissociable from tasks designed to test individual components of executive function. Thus, it is possible that harmonization exercises ones ability to simultaneously coordinate individual components, and improves ones ability to do so outside of a musical context, such as during planning or problem-solving. However, harmonic ability did not moderate the relationship between musical experience and planning/problem-solving in the current study. Therefore, a plausible alternative explanation for these findings could be that high scoring participants had pre-existing advantages in planning and problem-solving, which made them more successful at the harmonization task. This is consistent with reports of pre-existing differences between musically trained and untrained participants accounting for apparent advantages in cognition (Trimmer and Cuddy, 2008; Schellenberg, 2011; Swaminathan et al., 2017). Indeed, whether or not the observed effects were caused by the ability to harmonize can only be assessed by studies with random assignment to harmony training, active controls, and controls for confounds such as IQ and socioeconomic status. The current study provides a preliminary investigation of a relationship between harmony and executive function, but is limited by its lack of control for confounds and randomization.

The current findings are consistent with reports that musical experience is related to enhanced planning and problem-solving (Drake and Palmer, 2000; Berti et al., 2006; Sachs et al., 2017), but not consistent with reports of enhanced working memory and inhibitory control (Moreno et al., 2009, 2014; George and Coch, 2011; Slater et al., 2018). This discrepancy may be due in part to differences in the developmental stages of participants in the current study compared to participants in other studies (Moreno et al., 2009, 2014; Slater et al., 2018). Discrepant outcomes may also be due in part to differences in the tasks used to measure executive function (George and Coch, 2011). In addition, recent evidence indicates that rhythm training is selectively associated with inhibitory control (Slater et al., 2017, 2018; Frischen et al., 2019), suggesting that specific elements of musical training are related to specific cognitive outcomes. In the current study, musically experienced participants were recruited from harmony courses and it is possible that musically trained individuals with strong planning and problem solving abilities self-select for further harmony training. Furthermore, participants in the current study were young adults. It is possible that musically experienced participants may have shown music-related cognitive advantages for certain processes earlier in childhood, and musically inexperienced participants have since caught up. Taken together, the current findings add to the growing body of evidence that musical experience is associated with advantages in higher order cognitive processes. The differences in findings in current and previous reports highlight the need for systematic evaluation of types of musical experience and their relationships to cognitive processes across the lifespan.

A methodological contribution of the current study is demonstrating that a scale of music training has advantages over the categorical assignment of participants to groups of musicians and non-musicians. First, there are vast individual differences in levels of music training, and use of a scale permits investigation of a range of experience that better represents the population. A musicianship scale also allows for the reduction of several aspects of musical experience to a single value while maintaining fidelity to the experiences that contribute to musicianship. In addition, aspects of music training that are included in the scale are assessed for reasonable factorability, thus justifying use of items in a single scale. Previous efforts have been made to quantify musicianship, and with great success. For example, the Goldsmith Musical Sophistication Index distills a thorough and comprehensive overview of music listening behavior, innate musicality, musical experience, music training, and perceptual abilities into a single value (Müllensiefen et al., 2014). However, the current study was primarily concerned with music training, and the scale variable used was comprised of training-related items that loaded onto a single factor.

The current study extends previous reports that music training is related to improvements in executive function and provides evidence of a relationship between harmonization and planning and problem solving. Harmonization practice may facilitate coordination of individual components of executive function resulting in general enhancement of planning and problem-solving skills. Alternatively, those with high planning and problem-solving abilities may be predisposed to better harmonization. Future studies should investigate causality by randomly assigning participants to harmony training groups with active controls, and controlling for confounds such as intelligence and socioeconomic status. Overall, the current findings provide initial evidence of a relationship between harmony training and executive function.

## Data Availability Statement

The raw data supporting the conclusions of this article will be made available by the authors, without undue reservation.

## Ethics Statement

The studies involving human participants were reviewed and approved by the Tulane Institutional Review Board. The patients/participants provided their written informed consent to participate in this study.

## Author Contributions

JW and PC conceived of the presented idea and designed the study. BJ designed the harmony assessment and contributed to the theoretical foundation of the presented idea. JW carried out all data collection procedures under the supervision of PC. JW and DC conceived of and conducted all listed analyses. JW and PC wrote the manuscript in consultation with BJ and DC. All authors contributed to the article and approved the submitted version.

## Conflict of Interest

The authors declare that the research was conducted in the absence of any commercial or financial relationships that could be construed as a potential conflict of interest.

## Publisher’s Note

All claims expressed in this article are solely those of the authors and do not necessarily represent those of their affiliated organizations, or those of the publisher, the editors and the reviewers. Any product that may be evaluated in this article, or claim that may be made by its manufacturer, is not guaranteed or endorsed by the publisher.
